# Stroke survivors’ long-term QALY-weights in relation to their spouses’ QALY-weights and informal support: a cross-sectional study

**DOI:** 10.1186/s12955-017-0724-7

**Published:** 2017-07-25

**Authors:** Josefine Persson, Lars-Åke Levin, Lukas Holmegaard, Petra Redfors, Katarina Jood, Christina Jern, Christian Blomstrand, Gunilla Forsberg-Wärleby

**Affiliations:** 1 Department of Clinical Neuroscience, Institute of Neuroscience and Physiology, the Sahlgrenska Academy at the University of Gothenburg, Gothenburg, Sweden; 2 Health Metrics, the Sahlgrenska Academy at University of Gothenburg & Centre for Health Economics (CHEGU), University of Gothenburg, Box 414, 405 30 Gothenburg, Sweden; 3 Department of Medical and Health Science, Linköping University, Linköping, Sweden; 4 Department of Clinical Pathology and Genetics, Institute of Biomedicine, the Sahlgrenska Academy at the University of Gothenburg, Gothenburg, Sweden; 5 Stroke Centre West the Sahlgrenska Academy at the University of Gothenburg, Gothenburg, Sweden

**Keywords:** Stroke, Spousal informal support, Health-related quality of life, Quality-adjusted life-years, Dyadic perspective.

## Abstract

**Background:**

Healthcare interventions that have positive effects on the stroke survivors’ health-related quality of life (HRQoL) and quality-adjusted life-years (QALYs) might also have positive effects for their spouses in terms of improved HRQoL and/or reduced spousal informal support. However, knowledge about stroke survivors’ HRQoL and QALY and the consequences for their spouses’ HRQoL and QALY is limited. Therefore, the aim of this study was to describe the HRQoL and QALY-weights in dyads of stroke survivors in comparison with dyads of healthy controls, and to study the relationship between the stroke survivors’ QALY-weights and consequences for spouses in terms of QALY-weight and annual cost of informal support, using a long-term perspective.

**Methods:**

Data on stroke survivors, controls, and spouses were collected from the seven-year follow-up of the Sahlgrenska Academy Study on Ischemic Stroke (SAHLSIS). HRQoL was assessed by the SF-36, and the preference-based health state values were assessed with the SF-6D. The magnitude of the support was assessed with a study specific time-diary. An ordinary least squares (OLS) regression was used to estimate the association between stroke survivors’ and spouses’ QALY-weights. A two-part econometric model was used to estimate the association between stroke survivors’ QALY-weights and the time spent and cost of spouses’ informal support.

**Results:**

Cohabitant dyads of 248 stroke survivors’ aged <70 at stroke onset and 245 controls were included in the study. Stroke survivors had lower HRQoL in the SF-36 domains physical functioning, physical role, general health, vitality (*P* < 0.001), and social functioning (*P* = 0.005) in comparison with their cohabitant spouses. There was no significant difference in HRQoL for the dyads of controls. The results from the regression analyses showed that lower QALY-weights of the stroke survivors were associated with lower QALY-weights of their spouses and increased annual cost of spousal informal support.

**Conclusion:**

Our results show that the QALY-weights for stroke survivors had consequences for their spouses in terms of annual cost of spousal informal support and QALY-weights. Hence, economic evaluation of interventions that improve the HRQoL of the stroke survivors but ignore the consequences for their spouses may underestimate the value of the intervention.

## Background

The caregivers’ role is important in influencing the outcome after stroke within a mutual dyadic relationship. Stroke survivors living with their family or spouse arrive earlier at the hospital, receive more thrombolytic therapy, are more likely to return home [1], and receive more anticoagulants as secondary prevention [2], compared to stroke survivors living alone. Previous studies showed that stroke survivors’ living alone predicted mortality after stroke [3], which was especially true for male stroke survivors in the long-term perspective [4]. The caregiver’s characteristics also have an impact on the outcome of the stroke survivors. Caregivers’ depression was associated with lower scores of stroke survivors’ physical function and communication at 4 months after stroke onset, and social participation and mood at 12 months after stroke onset [5]. Results from the ASPIRE-S study [6] showed that, six-months after stroke onset, caregiver anxiety was predicted by the stroke survivor’s anxiety, depression and cognitive impairment, and caregiver depression was predicted by the stroke survivor’s anxiety and depression. The association between health-related quality of life (HRQoL) for dyads of stroke survivors has previously been studied in a short-term perspective of four and 16 months after stroke onset [7]. The results showed that there was no significant difference in the bodily pain, emotional role, and mental health between the dyads after 4 months and no difference in bodily pain and mental health after 16 months. The association between the dyads’ depression and HRQoL has, however, not been studied in a long-term perspective.

Spouses of stroke survivors experience a negative impact on their HRQoL during the first 2 years after the stroke onset [8], and we have shown that such a negative impact was also present 7 years after stroke onset [9]. In these previous studies, the impact on spouses and caregivers’ HRQoL was studied in relation to objective stroke-related outcome, such as functional status and emotional variables. However, there is a lack of knowledge concerning whether stroke survivors’ self-perceived physical, general, and mental health has consequences for their spouses that would be relevant for economic evaluations with a societal perspective. The relevant consequences in this perspective would be to show whether a health care intervention has an impact on the cost of informal support and on relatives’ health [10]. Quality-adjusted life-years (QALY) is the recommended health outcome measure in economic evaluations [11, 12]. A QALY is a generic measure that combines health status, often called QALY-weights, and time in the same outcome measure. However, knowledge about relative’s QALY-weight is in general limited [13]. We have previously shown that the spouses’ HRQoL were associated with measures of global function of the stroke survivors [9]. Furthermore, the quantity of informal support has also been shown to be associated with the stroke survivors’ functional ability [14], motor and cognitive function [15] and their stroke-related health problems [16]. We have recently shown that the spousal support and cost [17] and QALY-weight [18] were associated with measures of global function of the stroke survivors. However, it remains to be shown whether there is an association between the stroke survivors’ QALY-weights and the informal support provided by their spouses.

Hence, the aim of this paper were [1] to describe the HRQoL and QALY-weights in dyads of stroke survivors in comparison with dyads of healthy controls, [2] to study the relationship between QALY-weights within dyads of stroke survivors, and [3] to study the relationship between the stroke survivors’ QALY-weights and the annual cost of spousal informal support, using a long-term perspective.

## Method

### Subjects

The study population originated from the Sahlgrenska Academy Study on Ischemic Stroke (SAHLSIS) [9, 19]. The SAHLSIS database comprised 600 consecutively included patients diagnosed with stroke before the age 70, recruited from four stroke units in the Region Västra Götaland between 1998 and 2003. The patients were age, sex, and geographically matched with 600 controls. Residents from Gothenburg, older than 30 years, were selected randomly from a group of participants in a population-based health survey [20]. Residents from Skövde and Borås as well as controls younger than 30 years, were collected from the Swedish Population Register. All controls were evaluated at a face to face visit with a study nurse. The medical history was obtained using a structured questionnaire, and all controls were underwent investigation with an electrocardiography (ECG). Participants with a history of stroke, coronary, or peripheral artery disease (doctors’ diagnosis), or sign of ischemic heart disease on resting ECG according to the Minnesota code were excluded.

**Fig. 1:**
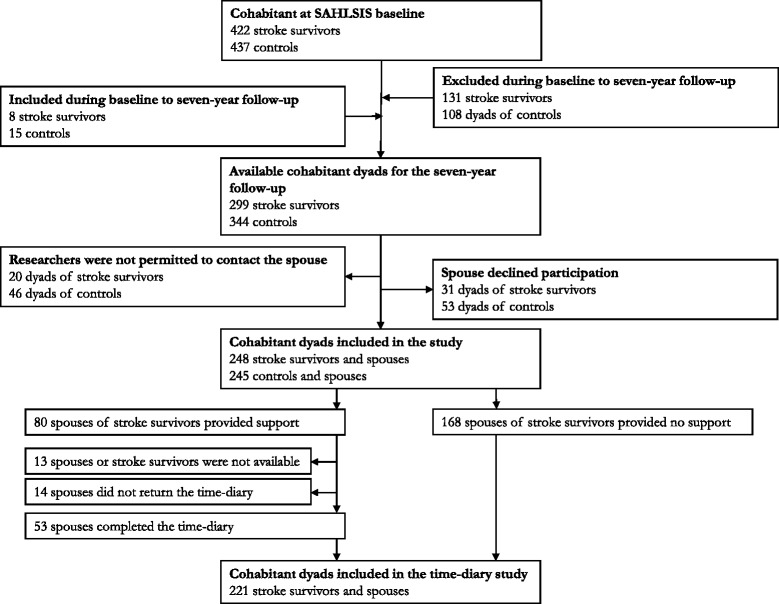
Flowchart of study population

For the seven-year follow-up, the patients and controls were invited to respond to a questionnaire regarding background variables and self-assessed instruments concerning health issues. Stroke survivors who were recruited at the Sahlgrenska University hospital were also invited to visit the research nurse and physician. Cohabitant spouses and partners of both stroke survivors and controls were requested to respond to a questionnaire regarding background variables and a self-assessed instrument for HRQoL. The recruited participants and drop-out rates have been presented in detail elsewhere [9]. In brief, at SAHLSIS baseline, 422 stroke survivors and 437 controls were cohabitant. In the seven-year follow-up, 299 stroke survivors and 344 controls were cohabitant, whereof 248 spouses of stroke survivors and 245 spouses of controls were recruited to the study (Fig. 1).

The stroke survivors who were lost from baseline to the seven-year follow-up had poorer global disability measured with the modified Rankin Scale (mRS) (*P* < 0.001), and more males than females were lost in the follow-up. During the period from baseline to the seven-year follow-up, 16.5% of the stroke survivors included in this study had a recurrent stroke. Of the controls, 1.6% had a stroke during the period from baseline to the seven-year follow-up.

Seventeen percent of the cohabitant stroke survivors, and 29% of the controls at the seven-year follow-up refused contact with their spouse, or the spouse declined participation in the study. There was no difference in age or sex between the stroke survivors or controls whose spouses were recruited to the study, compared to those whose spouses were not included in the study.

The data collection of informal support was made in a second step and of the 80 spouses that reported in the seven-year questionnaire that they provided support to their partner, 67 spouses were available for data collection of quantifying informal support in a study-specific time-diary, and 53 full-filled the data collection. Thus, the analysis concerning time spent and annual cost of informal support is based on 53 spouses reporting informal support and 198 spouses reporting no informal support, which provided a total study population of 221 dyads (Fig. 1). The recruited participants and drop-out in the time-diary study have been presented in detail elsewhere [17].

The recruited spouses in the time-diary study (*n* = 53), did not differ concerning spouses’ age, sex, occupational status, level of education, or the global disability of their stroke surviving partner as measured with the mRS, compared to the drop-outs (*n* = 27).

### Assessments and data collection

Sociodemographic data about the study population was collected from the SAHLSIS database. HRQoL was assessed using the Short Form-36 (SF-36) questionnaire (version 1) in an approved Swedish version [21]. The SF-36 consists of eight domains: physical function, physical role, bodily pain, general health, vitality, social functioning, emotional role, and mental health. An algorithm developed by Brazier et al. [22] was used to derive a preference-based measure of health from the SF-36 into the six-dimensional health state classification, i.e. SF-6D. The SF-6D six domains are physical functioning, role participation (combined role-physical and role-emotional), social functioning, bodily pain, mental health, and vitality. Each dimension has between four to six response levels, resulting in 18,000 different health states. Each health state has been weighted directly or indirectly using the standard gamble method on a random sample from the general population in the UK.

**Fig. 2:**
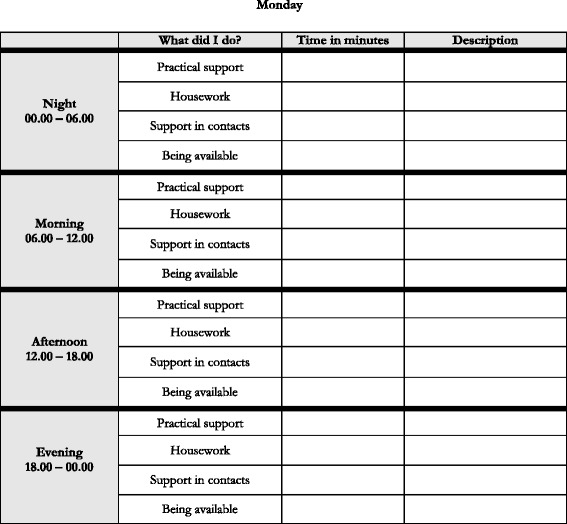
The time-diary

Data regarding quantity of informal support by the spouses of stroke survivors were collected by a study-specific time-diary. The spouses reported their informal support during one for them normal week with regard to four different categories of support, i.e. practical support, housework, support in contacts and being available (Fig. 2). In the analyses, the categories pertaining to practical support, housework, and support in contacts were aggregated into one category, i.e. practical support. A limit of 24 h were used in the analysis.

### Cost analyses

The cost of informal support was valued according to the opportunity cost method [23], i.e. the time spent on informal support was valued as the person’s best alternative use of the time (on work or leisure). Loss of production was valued by the human capital approach [23]. An hourly estimation of loss of production, including payroll taxes, of €20 (exchange rate 0.10 from € to SEK) was used [24]. Due to lack of data concerning whether the spouses reduced their working time to provide informal support, we valued the informal support as leisure time with a rate of €7, i.e. 35% of the hourly loss of production [25]. Joint production was considered for the category “being available”, i.e. when the spouses themselves benefited from the provided informal support. Hence, support as being available was valued at 50% of the leisure time, i.e. an hourly rate of €3.5. The annual cost of informal support was estimated by an extrapolation from the weekly reported support in the time-diaries.

### Statistical analyses

The variable distribution was presented as mean and standard deviation (SD) for continuous variables and as number and percentage for categorical variables. All significance tests were two-sided and conducted at the 5% significance level. The Wilcoxon signed rank test was used to test the statistical difference within the dyads of stroke survivors and dyads of controls.

To investigate the relationship between the QALY-weights of the dyads of stroke survivors, an ordinary least squared (OLS) regression was used. The dependent variable was the spouses’ QALY-weights. The stroke survivors’ QALY-weights were included as an independent variable (model 1), while model 2 were adjusted for spouses’ age, sex, educational level, and occupational status.

To investigate the relationship between the time spent and annual costs of spouses’ informal support and the stroke survivors’ QALY-weights, we used a two-part model [26] due to a large proportion of the spouses reporting that they provided no informal support. This model has been adopted for other studies estimating cost of informal care [16]. The first part of the jointly estimated two-part model was a binary choice model for estimating the probability of observing a positive outcome, and the second part was a regression model based on the observations with positive outcomes. The chosen approach was a logit for the first part and for the second part and an ordinary least squares (OLS) with the natural logarithm (ln) of the outcome variable, i.e. ln(hours) and ln(cost). For the retransformation from the ln-scale to the raw cost scale a Duan Smearing Approach was used [27]. The standard errors and confidence intervals were estimated with percentile bootstrap with 1000 replications. The dependent variables were time spent and annual cost of practical support and support by being available. The stroke survivors’ QALY-weights were included as an independent variable, and the model was adjusted for spouses’ age, sex and occupational status. The results for each part of the model were combined to yield a predicted estimate of the hours per day and annual cost of practical support and support by being available for each of the stroke survivor’s QALY-weights.

All the analyses were carried out in the STATA statistical software (version 14, College Station, TX, USA). For the two-part model, the “tpm” command was used and the “margins” command was used for predictions.

## Results

The population of this study consisted of 248 cohabitant dyads of stroke survivors and 245 cohabitant dyads of healthy controls. The mean ages of the spouse and the stroke survivors were 62 (range: 21–82) and 64 (range: 18–77), respectively, and 66% and 34% were females, respectively (Table 1). The study population for the analyses of informal support consisted of 221 dyads of stroke survivors. The mean ages of the spouse and the stroke survivors were 62 (range: 21–82) and 62 (range: 26–77), respectively, and 66% and 34% were females, respectively.

**Table 1:** Demographic features of the sample

	Spouses of stroke survivors (%) (*n* = 248)	Stroke survivors (%) (*n* = 248)	Spouses of controls (%) (*n* = 245)	Controls (%) (*n* = 245)
Mean age, y (SD)	63 (11)	64 (11)	64 (9)	65 (9)
Female sex	162 (65)	85 (34)	161 (66)	84 (34)
Education				
Secondary or less	96 (39)	92 (37)	71 (29)	83 (34)
High school	77 (31)	87 (35)	89 (36)	87 (36)
University	75 (30)	68 (28)	85 (35)	74 (30)
Occupation				
Employed fulltime	73 (29)	30 (12)	58 (24)	72 (30)
Retired fulltime	122 (49)	148 (60)	132 (54)	145 (56)
Other^a^	53 (21)	70 (28)	55 (22)	28 (14)
Household				
Children <18 at	28 (11)		22 (9)	
Support in home				
Informal support^b^	80 (32)		8 (3)	
Formal support^c^		25 (10)		0 (0)

^a^Other: Employed part-time, retired part-time, unemployed, sick leave and being a student

^b^Informal support: Self-reported information from the spouse concerning whether they provided informal support to their partner

^c^Formal support: Home care, personal assistant, or living at nursing home

In a long-term perspective of 7 years after stroke onset, there were significant differences between the stroke survivors and their spouses in the SF-36 domains concerning physical functioning, role physical, general health, vitality, social functioning and also in their QALY-weights. However, for the controls and their spouses, there were no significant differences between any of the SF-36 domains, nor the QALY-weights (Table 2). The HRQoL for stroke survivors in comparison with the controls was different for all the SF-36 domains, (*P* < 0.001, bodily pain *P* = 0.015). Mean SF-6D QALY-weight for spouses and stroke survivors were 0.75 (range: 0.44–0.94) and 0.70 (range: 0.30–0.94). Mean SF-6D QALY-weight for spouses and controls were 0.77 (range: 0.43–0.94) and 0.78 (range: 0.48–0.94).

**Table 2:** Health-related Quality of Life and SF-6D QALY-weights for dyads of stroke survivors and dyads of controls

	Spouses of stroke survivor	Stroke survivor	Mean difference	Spouses of controls	Controls	Mean difference
Physical functioning	84 (21)	67 (30)	17 (31)***	87 (18)	87 (16)	0 (21)
Role, physical	79 (36)	65 (41)	14 (49) ***	86 (32)	86 (31)	0 (42)
Bodily pain	72 (27)	71 (29)	1 (36)	76 (26)	79 (24)	3 (35)
General health	72 (23)	63 (23)	9 (28)***	76 (21)	78 (18)	2 (24)
Vitality	66 (24)	57 (25)	8 (30)***	73 (22)	76 (19)	3 (25)
Social functioning	87 (21)	81 (25)	6 (31)**	91 (19)	92 (17)	1 (23)
Role, emotional	81 (35)	80 (36)	0 (49)	90 (27)	93 (23)	3 (31)
Mental health	78 (20)	76 (20)	2 (25)	84 (16)	86 (15)	2 (19)
SF-6D QALY-weight	0.75 (0.12)	0.70 (0.12)	0.05 (0.15)***	0.77 (0.11)	0.78 (0.10)	0.01 (0.14)

Mean with standard deviations in parenthesis

Wilcoxon signed ranks test with following level of significance: ***1%, **5%, *10%

The results from the OLS regression showed that higher QALY-weights of the stroke survivors were associated with higher QALY-weights of their spouses (*P* < 0.001). When adjusted for spouses’ own demographic features, the association between the dyad’s QALY-weights was still significant (*P* = 0.002). Lower own QALY-weights were also found among the spouses who were retired, unemployed, on sick leave or part-time employed (Table 3).

**Table 3:** Estimating spouses’ QALY-weights as a function of patients’ QALY-weights with an ordinary least squared regression

	Model 1		Model 2	
	ß	*p*-value	ß	*p*-value
QALY-weight	0.205	<0.001	0.187	0.002
Spouses’ age			-0.001	0.230
Spouses’ sex				
Male^a^			0.008	0.623
Education				
High school^b^			-0.013	0.454
University^b^			-0.011	0.550
Occupation				
Retired^c^			-0.060	0.010
Other^c^			-0.064	0.002
Constant	0.607	<0.001	0.680	<0.001
R squared	0.048		0.142	
Observations	242		242	

The regression analyses were based on a sample of 242 due to missing SF-6D index for 3 stroke survivors and 3 spouses

Other: Employed part-time, retired part-time, unemployed, sick leave and being a student

Following references are used:

^a^Females

^b^Secondary school or less

^c^Employed

**Fig. 3:**
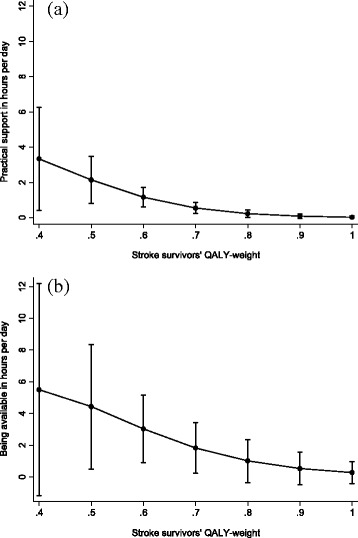
Spouses’ informal support in practical support (**a**) and being available (**b**) in mean hours per day per stroke survivors’ QALY-weight adjusted for formal support and spouses’ occupational status and including 95% CI error bars estimated by percentile bootstrap with 1000 replications

The spousal informal support in hours per day and annual costs was estimated as a function of the stroke survivors’ QALY-weight with a two-part econometric model. Figure 3, illustrates the predicted estimates of hours of practical support and being available in relation to the stroke survivors’ QALY-weights. Lower QALY-weight of the stroke survivor predicted more time of practical support and support by being available. For QALY-weights of ≥0.8, the associations with the time spent of practical support and support by being available were no longer significant. Similar results were shown for the annual cost of informal support, where for QALY-weights ≤0.8, the annual cost of practical support and support by being available gradually increased with lower QALY-weights of the stroke survivor (Table 4).

**Table 4:** Annual cost of spouses’ practical support and being available per stroke survivors’ QALY-weight. Costs are presented in € (2015)

Stroke survivors QALY-weight	Practical support	*P*-value	Being available	*P*-value
0.40	9009 (1339–16,680)	<0.021	6697 (-1581–14,976)	0.113
0.50	5787 (2233–9341)	<0.001	5398 (574–10,221)	0.028
0.60	3134 (1673–4594)	<0.001	3697 (1091–6305)	0.005
0.70	1460 (602–2318)	<0.001	2225 (293–4156)	0.024
0.80	609 (12–1205)	0.046	1230 (-340–2859)	0.139
0.90	233 (-112–578)	0.185	648 (-578–1874)	0.300
1.00	85 (-85–254)	0.328	334 (-498–1165)	0.432

Costs are adjusted for formal support and spouses’ occupational status, with 95% confidence intervals estimated by percentile bootstrap with 1000 replications

According to the output from the two-part regression model, the first part showed that the stroke survivors’ QALY-weights predicted their spouses’ practical support (*P* < 0.001) and support by being available (*P* < 0.001). However, in the second part of the model, the stroke survivors’ QALY-weights were not significantly associated with the spouses’ practical support and support by being available. Hence, according to our data, it was the fact that the spouses provided informal support, and not the time spent on informal support, that was the driving factor underlying the significant results regarding the post-estimates of time and annual cost of informal support per QALY-weight. The post-estimates of time spent on support and annual cost of informal support for stroke survivors with QALY-weight of 0.4 were not significant. This was probably due to few observations (*n* = 6) with QALY-weight 0.4 and wide distribution in the category “being available” (range: 0–24 h per day).

## Discussion

The main finding of this study is that lower QALY-weights of the stroke survivors were associated with their spouses’ lower QALY-weights and higher cost of informal support. Economic evaluation of a health care intervention that improves the HRQoL of the caregiver or reduces the carer time, underestimate the value of the intervention if those effects are excluded [28]. A systematic review concluded that the literature has not sufficiently taken into account the theories, guidelines and methods that support the inclusion of informal care in applied economic evaluations [29]. However, it can be noted that the studies in this review that incorporated informal support seemed to have an impact on the cost-per-QALY estimate. Our findings indicate that from a societal perspective, economic evaluations of health care interventions for the stroke survivors that improve their QALY-weights should also incorporate the spousal effects to capture an essential part of the total effect in dyads of stroke survivors.

A previous study by Dixon et al. [30] showed that the EQ-5D score for a large cohort of patients with various diseases was associated with increased carer time. Although this study also showed an association between carer time and QALY-weights, the study objectives differed from ours in several ways. First, the study population consisted of patients with various diseases in a short-term perspective, while we studied dyads of stroke survivors in a long-term perspective. Second, the questions concerning carer time from friends and relatives were answered retrospectively by the patients themselves, while we used a time-diary answered prospectively by the spouses to highlight the subjective perceptions of informal care by the spouses themselves and to prevent recall bias [31]. Third, their study used the EQ-5D while we used the SF-6D instrument, and these instruments have in previous studies yielded different results [32].

According to a recent systematic review [33], the average weekly care time for stroke survivors was 23.98 h. This approximately corresponds to the spousal weekly support provided to stroke survivors with a QALY-weight of 0.6, according to our data, while a QALY-weight of 0.7 corresponds to a weekly spousal support of 13 h and a QALY-weight of 0.5 corresponds 41 h of spousal support per week. According to this systematic review [33], the average unit cost per hours of informal support valued with the opportunity cost method was €10.14, which is somewhat higher compared to our shadow price for loss of leisure time. The appropriate shadow price to use for leisure time, if any, is debated in the literature [31]. We chose to use a shadow price for leisure that corresponded to 35% of loss of production, a method that originates from the transport sector and has been used in other studies [25, 34–37].

The study by Dixon et al. [30] also investigated the association between the EQ-5D scores of patients with Alzheimer’s and their primary caregivers. In contrast to our results, they did not find a significant association between the patients’ QALY-weights and those of their caregivers. One explanation for this might be that Alzheimer’s disease and stroke have different courses. Furthermore, Dixon et al. included primary caregivers whereof 52% were spouses, while we included solely spouses. The rationale for highlighting the consequences for this particular subgroup of caregivers was that the impact on HRQoL might be different for spouses who were cohabiting with the stroke survivors compared with other non-cohabitant family members and friends [38].

Our findings show that dyads of stroke survivors report similar levels in the domains of bodily pain, emotional role, and mental health. These results are in line with a previous study by Jönsson et al. [7] of caregivers and stroke survivors in a 4 month follow-up after stroke onset. However, in the second follow-up, 16 months after stroke onset, the stroke survivors reported an improvement in the emotional role-domain compared to their caregivers. According to Jönsson et al., this may be due to the stroke survivors’ better adaptation to the new situation compared to their caregivers. Furthermore, a study of dyads of patients with chronic heart failure shows that patients reported lower HRQoL in all the SF-36 domains compared to their spouses, except in the mental health domain [39]. Similar to our findings, the patients’ QALY-weights (0.63) measured with the SF-6D were also significantly lower in comparison to their spouses’ QALY-weights (0.78). Previous studies have indicated that depression seems to have a negative impact on the dyads in both directions [5, 6, 40]. Further longitudinal studies are needed to fully understand the dyadic perspective of mental illness of stroke survivors and their spouses. However, our results indicate that there could be a need of targeting interventions to improve both the stroke survivors’ and their spouse’s mental health after a stroke and also in a long-term perspective.

The advantage of studying this well-documented population with consecutively included stroke survivors is that it highlights a younger population, many with responsibility for family and working life in a long-term perspective, whereas most studies have focused on an older population in a shorter time-perspective. The findings in this study should however not be generalised neither to a short-term perspective nor to other groups such as older dyads of stroke survivors or other family members, due to two reasons. Firstly, spouses of midlife stroke survivors often also have responsibilities for their family and an own professional life [41]. Thus, younger spouses may experience a greater conflict between their regular daily family and household chores, working lives, and the support provided to their partner, in comparison to older spouses or to other caregivers such as children and friends. Secondly, given that younger stroke survivors have longer survival time, further enhanced by the secular trend of decreasing risk of mortality [42], spouses must provide support to their partner over a longer period of time compared to older stroke survivors. Moreover, the spouses prospectively reported the quantity of their provided informal support in a study-specific time-diary, a method that minimises the recall bias [31]. However, the study has also some limitations. The sample that reported the time spent on informal support was small, with a relatively wide distribution, especially in the category “being available”. This might be one of the reasons why the stroke survivors’ QALY-weights were not significantly associated with the spousal time spent on informal support. Instead, the support that were provided by the spouses, was the driving factor for the post-estimates from the two-part model. Due to the small sample the generalizability of the result might be limited due to possible type II errors. In the two-part model we used the percentile bootstrap as a resampling method for obtaining the 95% confidence intervals of a larger bootstrapped sample to handle the skew and small data. However, further research with a larger sample size is needed to confirm and elucidate our results. A further limitation was that we did not collect information regarding the medical history of the spouses, and could hence not adjust the analyses for the spouses’ own possible sickness or diseases. Moreover, we did not have longitudinal data for the spouses, but solely for the stroke survivors. Since many stroke survivors with poorer global disability at 3 months after stroke onset were lost to follow-up, the spouses’ reported consequences might underestimate the consequences at least with regard to those in a shorter time perspective. Further longitudinal data regarding spouses’ consequences need to be examined to achieve a more complete economic evaluation of health care interventions.

## Conclusion

The results in this study show that the QALY-weights for stroke survivors relate to their spouses’ consequences in terms of spousal time spent on informal support and spousal QALY-weights. Hence, economic evaluations of interventions that improve the HRQoL of the stroke survivors, but ignores to include the consequences for their spouses may underestimate the value of the intervention. Thus, the inclusion of spouses’ consequences in economic evaluations could have an impact on the cost-per-QALY estimates.

## Abbreviations

EQ-5D: EuroQol 5 dimension
HRQoL: Health-related quality of life
mRS: modified rankin scale
OLS: Ordinary least squares
QALY: Quality-adjusted life-years
SAHLSIS: Sahlgrenska Academy Study on Ischemic Stroke
SD: Standard deviation
SF-36: Short-Form 36
SF-6D: Short-Form 6 dimensions

## References

[1] Reeves MJ, Prager M, Fang J, Stamplecoski M, Kapral MK (2014). Impact of living alone on the care and outcomes of patients with acute stroke. Stroke.

[2] Sjölander M, Eriksson M, Asplund K, Norrving B, Glader EL (2015). Socioeconomic inequalities in the prescription of oral anticoagulants in stroke patients with atrial fibrillation. Stroke.

[3] Lindmark A, Glader EL, Asplund K, Norrving B, Eriksson M (2014). Collaboration Riks-stroke collaboration. Socioeconomic disparities in stroke case fatality - observations from Riks-stroke, the Swedish stroke register. Int J Stroke.

[4] Redfors P, Isaksén D, Lappas G, Blomstrand C, Rosengren A, Jood K (2016). Living alone predicts mortality in patients with ischemic stroke before 70 years of age: a long-term prospective follow-up study. BMC Neurol.

[5] Klinedinst J, Gebhardt M, Aycook D, Nichols-Larsen D, Uswatte G, Wolf S (2009). Caregiver characteristics predicts stroke survivors quality of life at 4 month and 1 year. Res Nurse Health.

[6] Atteih S, Mellon L, Hall P, Brewer L, Horgan F, Williams D (2015). Implications of stroke for caregiver outcomes: findings from the ASPIRE-S study. Int J Stroke.

[7] Jönsson AC, Lindgren I, Hallström B, Norrving B, Lindgren A (2006). Determinants of quality of life in stroke survivors and their informal caregivers. Stroke.

[8] Saban KL, Sherwood PR, DeVon HA, Hynes DM (2010). Measures of psychological stress and physical health in family caregivers of stroke survivors: a literature review. J Neurosci Nurs.

[9] Persson J, Holmegaard L, Karlberg I, Redfors P, Jood K, Jern C (2015). Spouses of stroke survivors report reduced health-related quality of life even in long-term follow-up: Results from Sahlgrenska Academy study on ischemic stroke. Stroke.

[10] Davidson T, Levin L (2010). Is the societal approach wide enough to include relatives? Incorporating relatives' costs and effects in a cost-effectiveness analysis. Appl Health Econ Health Policy.

[11] Socialstyrelsen. Nationella riktlinjer för sjukdomsförebyggande metoder 2011 - Hälsoekonomiskt underlag (Bilaga). [National guidelines for prevention methods 2011 - Health economic evidence]. Socialstyrelsen, Stockholm. 2011. Available from: http://www.socialstyrelsen.se/nationellariktlinjerforsjukdomsforebyggandemetoder/Documents/nr-sjukdomsforebyggande-halsoekonomisktunderlag.pdf. [Accessed: 16 Jan 2017].

[12] NICE. Guide to the methods of technology appraisal 2013. Available from: https://www.nice.org.uk/process/pmg9/chapter/foreword. [Accessed 16 Jan 2017].

[13] Wittenberg E, Prosser LA (2013). Disutility of illness for caregivers and families: a systematic review of the literature. PharmacoEconomics.

[14] Olai L, Borgquist L, Svärdsudd K (2015). Life situations and the care burde for stroke patients and their informal caregivers in a prospective cohort study. Uppsala J Med Sci.

[15] Tooth L, McKenna K, Barnett A, Prescott C, Murphy S (2005). Caregiver burden, time spent caring and health status in the first 12 months following stroke. Brain Inj.

[16] Hickenbottom SL, Fendrick AM, Kutcher JS, Kabeto MU, Katz SJ, Langa KM (2002). A national study of the quantity and cost of informal caregiving for the elderly with stroke. Neurology.

[17] Persson J, Levin L-Å, Holmegaard L, Redfors P, Svensson M, Jood K (2017). Long-term cost of spouses' informal support and its associastin with midlife stroke survivors' dependency. Brain and Behaviour.

[18] Persson J, Aronsson M, Holmegaard L, Redfors P, Stenlöf K, Jood K, et al. Long-term QALY-weights among spouses of dependent and independent midlife stroke survivors. Qual Life Res. 2017;epub ahead of print. 10.1007/s11136-017-1636-z PMC5655581 28664459

[19] Jood K, Ladenvall C, Rosengren A, Blomstrand C, Jern C (2005). Family history in ischemic stroke before 70 years of age: the Sahlgrenska Academy study on ischemic stroke. Stroke.

[20] Wilhelmsen L, Johansson S, Rosengren A, Wallin I, Dotevall A, Lappas G (1997). Risk factors for cardiovascular disease during the period 1985-1995 in Goteborg, Sweden the GOT-MONICA project. J Intern Med.

[21] Sullivan M, Karlsson J (1998). The Swedish SF-36 health survey III. Evaluation of criterion-based validity: results from normative population. J Clin Epidemiol.

[22] Brazier J, Roberts J, Deverill M (2002). The estimation of a preference-based measure of health from the SF-36. J Health Econ.

[23] Drummond M, Sculpher M, Torrance G, O'Brien B, Stoddart G (2005). Methods for the economic evaluation of health care programmes.

[24] Statistics Sweden. Average basic salary, monthly salary and women’s salary as a percentage of men’s salary by region, sector, occupation and sex. Available from: http://www.scb.se/. [Accessed: 16 Jan 2017].

[25] Johannesson M, Borgquist L, Jönsson B, Råstam L (1991). The cost of treating hypertension - an analysis of different cut-off points. Health Policy.

[26] Belotti F, Deb P, Manning WG, Norton EC (2015). Twopm: two-part models. Stata J.

[27] Duan N (1983). Smearing estimate: a nonparametric retransformation method. J Am Stat Assoc.

[28] Al-Janabi H, Flynn TN, Coast J (2011). QALYs and carers. PharmacoEconomics.

[29] Goodrich K, Kaambwa B, Al-Janabi H (2012). The inclusion of informal care in applied economic evaluation: a review. Value Health.

[30] Dixon S, Walker M, Salek S (2006). Incorporating carer effects into economic evaluation. PharmacoEconomics.

[31] van den Berg B, Brouwer WB, Koopmanschap MA (2004). Economic valuation of informal care. An overview of methods and applications. Eur J Health Econ.

[32] van Stel HF, Buskens E (2006). Comparison of the SF-6D and the EQ-5D in patients with coronary heart disease. Health Qual Life Outcomes.

[33] Oliva-Moreno J, Trapero-Bertran M, Pena-Longobardo LM, Del Pozo-Rubio R (2017). The valuation of informal Care in Cost-of-Illness Studies: a systematic review. PharmacoEconomics.

[34] Persson J, Ferraz Nunes J, Karlberg I (2012). Economic burden of stroke in a large county in Sweden. BMC Health Serv Res.

[35] Husberg M, Davidson T, Hallert E (2017). Non-medical costs during the first year after diagnosis in two cohorts of patients with early rheumatoid arthritis, enrolled 10 years apart. Clin Rheumatol.

[36] Jowett S, Bryan S, Mahe I, Brieger D, Carlsson J, Kartman B (2008). A multinational investigation of time and traveling costs in attending anticoagulation clinics. Value Health.

[37] Norlin JM, Elf JL, Svensson PJ, Carlsson KS (2010). A cost-effectiveness analysis of diagnostic algorithms of deep vein thrombosis at the emergency department. Thromb Res.

[38] Herlitz C, Dahlberg L (1999). Causes of strain affecting relatives of Swedish oldest elderly. A population-based study. Scand J Caring Sci.

[39] Ågren S, Evangelista L, Davidson T, Stromberg A (2011). The influence of chronic heart failure in patient-partner dyads--a comparative study addressing issues of health-related quality of life. J Cardiovasc Nurs.

[40] Grant JS, Clay OJ, Norman KL, Haley WE, Wadley VG, Perkins MM (2013). Does caregiver well-being predic stroke survivor depressive symptoms? A mediation analysis. Top Stroke Rehabil.

[41] Green TL, King KM (2007). The trajectory of minor stroke recovery for men and their female spousal caregivers: literature review. J Adv Nurs.

[42] Rosengren A, Giang KW, Lappas G, Jern C, Toren K, Bjorck L (2013). Twenty-four-year trends in the incidence of ischemic stroke in Sweden from 1987 to 2010. Stroke.

